# Response of water yield to silvicultural treatments in a temperate forest in northern Mexico

**DOI:** 10.1371/journal.pone.0291094

**Published:** 2023-12-15

**Authors:** José Carlos Monárrez González, Celia Lopez-Gonzalez, Marco Antonio Marquez-Linares, Gustavo Perez-Verdin

**Affiliations:** 1 Campo Experimental Valle del Guadiana, Instituto Nacional de Investigaciones Agropecuarias y Forestales, Durango, México; 2 CIIDIR Unidad Durango, Instituto Politécnico Nacional, Durango, México; Oregon State University, UNITED STATES

## Abstract

Forest management modifies tree cover, directly influencing various ecosystem services, such as water regulation. Evapotranspiration, water interception, surface runoff, stemflow, and throughfall are among those processes that depend on tree cover. The objective of this study was to evaluate the changes in water yield, defined as the difference between precipitation and evapotranspiration, after the application of silvicultural treatments in a temperate forest ecosystem in northern Mexico. Fifteen experimental plots were established in which the following treatments were applied: intensive management (clearcutting), semi-intensive management (selection, tree cutting of 59–61% of basal area), conservative management (selection, tree cutting of 29–31% of basal area), and no treatment (control group). Incident precipitation, throughfall, stemflow, net precipitation, interception, and surface runoff were analyzed. Results show that conservative management treatments increase water yield between 2 to 3.6 mm per m^-2^ ha^-1^ of tree basal area removed. Water flow distribution, in relation to the incident precipitation, ranged from 72.3 to 91.8% for throughfall, 0.2 to 0.4% for stemflow, 72.7 to 91.8% for net precipitation, 8.19 to 27.42% for interception or evaporation, and 0.54 to 1.93% for surface runoff. The conservative management treatment was the most viable alternative for increasing water yield without compromising timber harvesting and loss of soil due to hydric erosion.

## Introduction

Forest ecosystems have an essential role in the provision of ecosystem services. Forests supply fresh water and regulate other components of the hydrological cycle, such as surface runoff, infiltration, and groundwater storage, that directly affect people and support other functions of the ecosystem [1, 2]. Water yield is defined as the difference between precipitation and evapotranspiration, or the freshwater that reaches the surface soil, runs off or infiltrates into the subsoil [3, 4]. Water yield from rain depends, among other things, on vegetation cover, which determines the way precipitation is distributed [3–6]. Tree cover changes either by natural factors or the impact of human actions, such as forest fires, drought, diseases, as well as overgrazing and forest management practices [7]. Forest management involves practices and decisions aimed at the sustainable use of forest resources to improve the health of ecosystems and wellbeing of current and future generations [8]. These practices modify the structure, age, and species’ diversity in the forest [9], directly influencing the distribution of precipitation. Maintaining tree cover is also important for soil erosion control, reducing surface runoff, and favoring infiltration [10].

Rain distribution in a forested area can be subdivided into incident precipitation, interception, throughfall, stemflow, and surface runoff. Incident or total precipitation (*IP*) is the water released from clouds mostly in the form of rain [10]. Interception (*I*) is the amount of water that is retained by the canopy and evaporates during or after rainfall events [11–13]. Throughfall (*T*) is the fraction of water that directly reaches the forest floor through the canopy, but not through the stem [14, 15]. Stemflow (*SF*) is the part of the precipitation that is directed to the forest floor flowing down tree stems [10, 16]. Surface runoff (*SR*) refers to the rainfall that flows over the surface of the soil directly into nearby channels and bodies of water. Net precipitation (*NP*) is the amount of water that reaches the forest floor; it is a combination of throughfall and stemflow [17].

Studies regarding the impact of forests on water flow or yield have been mainly conducted in the USA and Europe. Worldwide, in dry forest ecosystems with rainfall gradients between 145 and 805 mm per year, the interception, throughfall, and stemflow represented approximately 24, 69.8, and 6.2% of precipitation, respectively [18]. In European forest ecosystems, composed mainly by species of the genera *Abies*, *Quercus*, *Pinus*, and *Fagus*, throughfall values reached 69 to 88% and stemflow between 0.7 to 12.3% [6, 19]. In experiments conducted at varying cutting intensities, throughfall was 83.8%, 66.7%, and 63% for high, moderate, and low intensity, respectively [20]. The long-term effect of patchwork clearing, uniform clearing, strip clearing, and removal of the undergrowth was assessed in forests in Australia. The authors concluded that water yield was greater in strip clearing, followed by patchwork clearing. They also found that as vegetation cover recovered, water yield decreased [21]. Hornbeck et al., [22], studied the immediate effects of tree removals in intensities between 13 and 93% of basal area in northeastern USA. They found that the increase of water yield was proportional to the reduction of basal area; nevertheless, the yield decreased 3 to 10 years after harvesting.

It is noteworthy that even though researchers in Mexico (and worldwide) are increasingly interested on the use of forest areas for the provision of water, there are few studies that assessed the impact of tree vegetation treatments on water yield. The paucity of information precludes the implementation of forest management practices that combine water regulation and timber production in the most productive ways, while at the same time minimizing the impacts on other ecosystem services, such as erosion control and biodiversity conservation. In northern Mexico, a few studies have been conducted to assess the relationship between temperate forest cover and surface runoff, stemflow, and throughfall [23, 24]. Even though they obtained consistent results, there is a need to continue investigating this relationship to improve forest management decision-making here and in other parts of the country.

In this study, we assessed the effect of various silvicultural treatments on water yield in a managed forest of the temperate ecosystem in northern Mexico. Our results will provide quantitative data on the importance of rainwater distribution in one of the largest, continuous areas of pine-oak forest in Mexico.

## Materials and methods

### Study area

**Fig 1:**
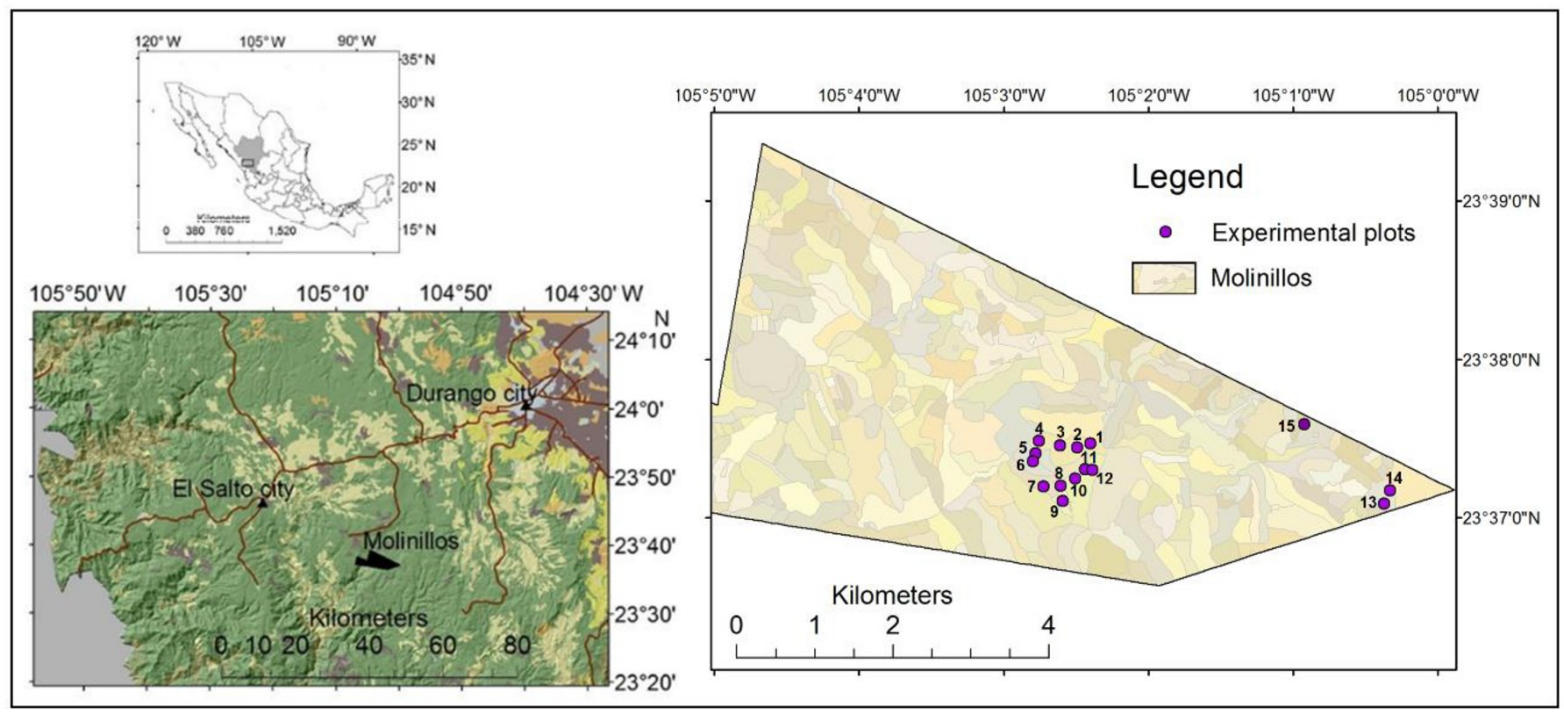
Location of the Molinillos property and the experimental plots to measure water yield components in Durango, Mexico. The type of silvicultural treatments for each plot are shown in Table 1 (Source: own information and Instituto of Geography and Statistics, INEGI).

This research was conducted in the Sierra Madre Occidental mountain range, northwestern Mexico. This mountain system was formed during two main geological periods of magmatic activity: the late Cretaceous and early Miocene, which produced a large silicic volcanic province [25]. The topography includes canyons, plateaus, and elevations between 2200 and 2800 m above sea level. The 2860-ha area is a private property known as “Molinillos”, which is located in the south of the Sierra Madre and in the state of Durango (Fig 1). Runoff flows toward the hydrographic system of the Acaponeta River basin and eventually to the Pacific Ocean. The vegetation is composed of native trees such as pines (57%), oaks (39%), other coniferous (3%), and broadleaved trees (1%). While forests are mostly naturally regenerated, a few areas (about 5% of total area and where clearcuts are applied) have been artificially reforested with native species [26]. The climate is humid and semi-cold, with a mean annual temperature of 10°C, ranging between -3°C and 18°C. Most of the precipitation comes in the months of July through September with an average of 800 mm per year [24]. To ensure the establishment of regeneration in clear-cuts, the forest management plan directs soil preparation activities such as subsoil ripping, soil erosion control using logging residues, and fencing against wildlife and domestic cattle. Landowners’ main objectives are to achieve sustainable management of forest resources, through international certification and application of good management practices, and promote ecotourism, hunting, and research and education [26]. Several research projects have been conducted in the area with the full support of the owners. Apart from the landowners’ interest to achieve better forest management decisions, the property has a permanent surveillance system, which facilitates the use and operation of the equipment and data collection. Commercial wood production is mostly done in pines (*Pinus teocote*, *P*. *durangensis*, *P*. *engelmanii*) and various species of oaks (*Quercus sideroxyla*, *Q*. *rugosa*, and *Q*. *convallata*). The area follows an authorized management plan in which the silvicultural treatments of individual tree selection, clearcutting, and forest thinnings are applied [26].

### Experimental design

To evaluate the effect of tree cutting intensities on water flow, in 2015 an experimental design was established with four treatments and two repetitions each. The treatments were applied in 15 circular plots with a total area of 2,500 m^2^ (28.21 m radius) and a usable area of 1,000 m^2^ (17.84 m radius) each. The treatments consisted of (1) intensive management (INT) that entailed clearcutting or removal of all trees; (2) semi-intensive management (SEMI) with selection and cutting around 60% of basal area (*BA)*; (3) conservative management (CON) with selection and cutting around 30% of *BA*; and (4) a control group with no treatment (NT) or no cutting. The assignment of treatments for each plot is shown in Table 1.

**Table 1:** Dasometric, ecological, and water characterization of research plots in temperate forests in Durango, Mexico.

	Plot number														
	1*	2*	3*	4	5*	6	7	8	9	10	11*	12*	13	14e*	15*
**Management treatment**	NT	SEMI	NT	SEMI	CON	CON	NT	CON	NT	SEMI	CON	SEMI	INT	INT	INT
***N* before clearing**	830	610	700	810	990	740	620	430	1070	670	420	570	430	790	1020
***BA* (m^2^ ha^-1^) before clearing**	32.38	24.13	25.01	31.85	30.68	17.84	16.95	11.03	18.49	18.67	25.17	13.64	13.49	18.03	24.94
**% Cutting intensity**	0	60.9	0	62.75	31.58	31.66	0	32.67	0	59.26	31.05	58.24	100	100	100
***BA* m**^**2**^ **ha**^**-1**^ **after clearing**	32.38	9.44	25.01	11.86	20.99	12.2	16.95	7.43	18.49	7.61	17.36	5.7			
**Normal diameter (cm)**	19.11	15.37	17.98	16.84	16.82	14.94	16.9	14.06	12.97	14.87	21.84	13.6	-	-	-
**Mean total height (m)**	12.98	9.83	11.65	11.38	12.58	8.68	10.25	8.23	8.8	8.73	12.2	8.46	-	-	-
**Tree volume (m**^**3**^ **ha**^**-1**^**)**	367.9	82.6	298	126.5	233.2	94.3	120.5	51.4	138.9	63.4	193.7	44.9	-	-	-
**Elevation (m)**	2357	2398	2350	2357	2358	2359	2439	2482	2441	2488	2447	2378	2598	2600	2612
**Aspect**	NW	NE	NE	NW	NW	SW	SW	SW	SW	NE	SE	SE	N	NW	W
**Slope (%)**	60	51	55	53	55	67	50	41	54	55	49	43	30	42	33
**H’**	1.08	1.45	2.02	1.92	1.32	1.81	1.59	1.44	1.26	0.89	1.75	1.88	1.08	1.45	2.02
**Λ**	0.52	0.69	0.69	0.46	0.69	0.82	0.84	0.77	0.83	0.81	0.74	0.81	0.52	0.69	0.69

* Plots with hydrological equipment and accessories. INT: intensive management (100% clearing of *BA*), SEMI: semi-intensive management (60% cutting of *BA*), CON: conservative management (30% cutting of *BA*), NT: no treatment. N: number of trees per ha, *BA*: basal area, H’: Shannon and Weiner diversity index, λ: Simpson’s diversity index.

Prior to the application of the silvicultural treatments, we collected information on site characteristics like slope, aspect, elevation, and soil texture, which are associated with surface runoff. Other dasometric variables (i.e., vegetation measurements) like the number of trees, normal diameter (or tree diameter at 1.30 m), height, age, and crown diameter were measured as well. Afterwards, these variables were used to calculate tree basal area, crown area (or canopy cover), and volume (Table 1). We also identified the taxonomic species of all trees to calculate the Shannon and Weiner and Simpson’s diversity indices. They were used to characterize the sites and compare the effects of the application between silvicultural treatments.

The slope of the research sites varied from 30% to 60%, while the terrain had mostly NW, SW, NE, SE, and W aspect gradients. Soil texture was predominantly sandy loam and soil deep varied from 0 to 50 cm. The main tree species were *Pinus duranguensis*, *P*. *teocote*, and *Quercus sideroxyla*. The tree volume averaged 67.75 m^3^ ha^-1^, 143.82 m^3^ ha^-1^, and 152 m^3^ ha^-1^ in the conservative, semi-intensive, and intensive management plots, respectively. Simpson (λ) and Shannon-Wiener (H’) diversity indices exhibited a medium to high diversity (Table 1). Once the silvicultural treatments were applied, we established the material and equipment to measure incident precipitation, throughfall, stemflow, and surface runoff. The assessment included the entire rainfall period, which typically comprises of 20–40 rainfall events, but useful data were taken from August to October 2016.

### Measurement of precipitation components

**Fig 2:**
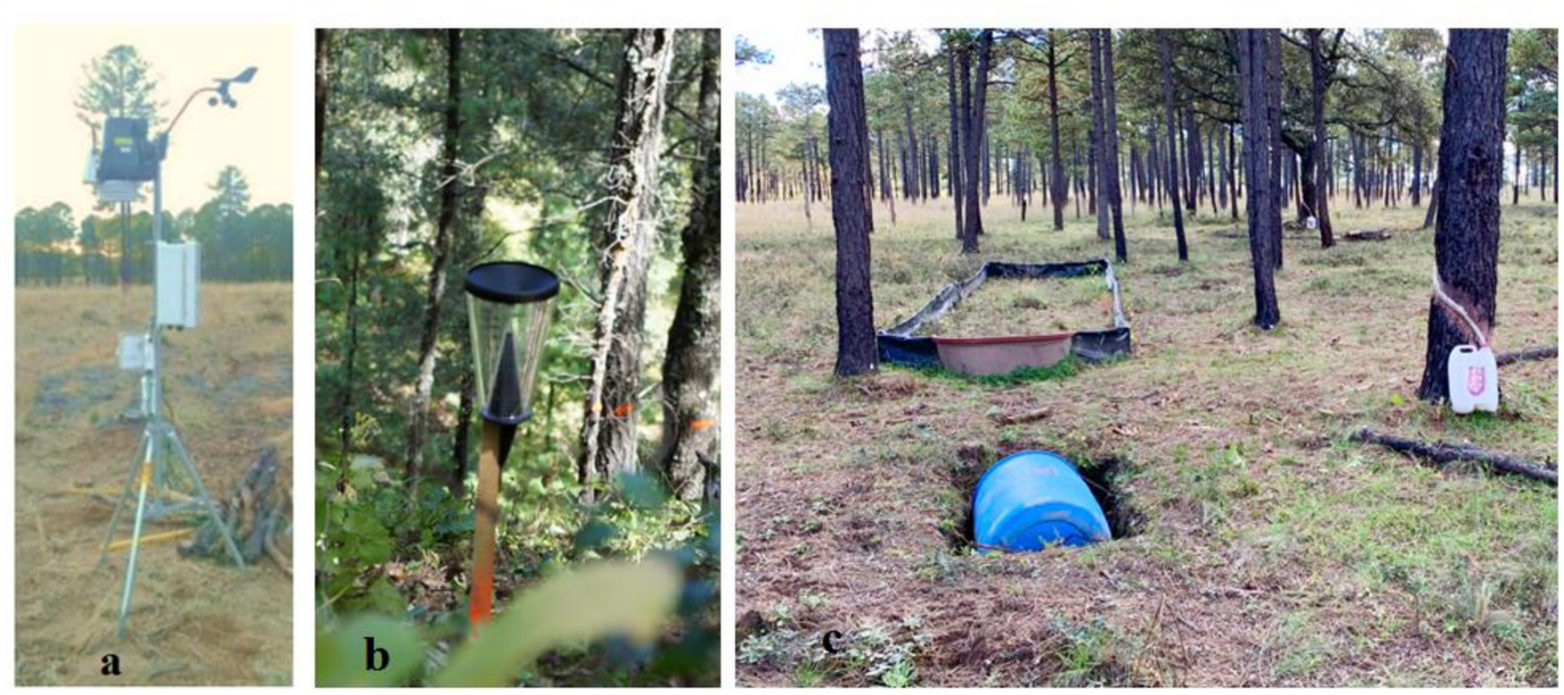
Measurement of precipitation components: a) A Vantage Pro Plus weather station in a clear stand to measure incident precipitation, b) A pluviometer measuring throughfall, c) A 16-m^2^ closed plot with a 200-l recipient capturing surface runoff and a recipient catching stemflow.

Precipitation components were measured during or after rainfall events. Incident precipitation (*IP*) was measured every 30 minutes using a Vantage Pro Plus weather station placed under an open sky next to the study area (Fig 2a). Due to economic and logistic issues, throughfall, stemflow, and surface runoff were measured in 8 out of the 15 plots; i.e., two for each treatment (Table 1). The rest of the plots were used to corroborate stand growth responses to the silvicultural treatments. Throughfall (*T*) was measured using four 70-mm pluviometers placed under the tree canopy of each experimental plot; three of them oriented at N, SE, and SW cardinal directions and at 8 meters from the plot center (Fig 2b). A fourth pluviometer was placed in a subplot that was intended to measure surface runoff (Fig 2c).

Stemflow (*SF*) was measured in 3 to 5 representative trees per plot, which were selected by genus and, if there was only one genus, by diameter class. The total number of selected trees was 32, of which 12 were oak trees (*Quercus* spp.), 12 were pine trees (*Pinus* spp.), and 8 were madrone (*Arbutus* spp) trees. The sampled trees were selected from plots having the semi-intensive, conservative, and control treatments. In each tree, a rubber hose was wrapped around the stem that directed the water flow to a 20 L tank (Fig 2c). Surface runoff (*SR*) was measured in 16-m^2^ (8x2 m) closed runoff subplots that directed water to 200-liter tanks (Fig 2c). This subplot was placed within the 1,000-m^2^ circular plot parallel to the surface’s slope. Data for *T*, *SF*, and *SR* were manually recorded after each rainfall event.

#### Data analysis

Dasometric, ecological, and hydrological characterization of the sites were conducted for each plot. We estimated *T*, *SF*, and *SR* coefficients by dividing each respective measurement by incident precipitation (*IP*). Interception *I* was estimated using the following equation [27]:

I=IP–T–SF, (1)

while net precipitation (*NP*) was estimated through:

NP=T+SF (2)

Net precipitation was used as a proxy for water yield estimation. Water flow partitioning was also differentiated by each silvicultural treatment. Values were handled in volume units (l m-^2^ and l ha^-1^) and later converted to depth equivalents (mm), following conventional units of water balance. Depth conversion for surface runoff was done using the volume of water captured in the 16-m^2^ closed plots. As for stemflow, depth conversion was done using regression models estimating the total water captured per genus, hectare. The exponential model for evaluating stemflow at tree level (*SF*), as a function of normal diameter (*ND*, measured in cm) and throughfall (*T*, measured in mm), was as follows:

$$SF=\beta_{1\;*}(ND^{\beta 2})\;*\;(T^{\beta 3})$$ (3)

where *SF* is the stemflow per tree and genus (liters per hectare), *β*_*1*_, *β*_*2*_, and *β*_*3*_ are the parameters of the model. Other linear and non-linear models that associate each rainfall component (throughfall, stemflow, and surface runoff) with dasometric variables (normal diameter, height, basal area, tree volume, and crown cover) were adjusted. The resulting models were then used to estimate rainfall components in those plots where no equipment and accessories were installed (see Table 1).

All regression models were adjusted using the Statistical Analysis System (SAS) software package (Draper & Smith, 1998). Selection of the best models was done based on the sum of square errors (SSE), goodness of fit of the mean squared error (MSE), the value and probability of F, R^2^ statistics, as well as the Student’s-t probability and confidence intervals of the estimators [28]. Normal distribution (Shapiro-Wilks’ test) and homoscedasticity (Levene’s test) were used to determine the use of a one-factor ANOVA or Kruskal-Wallis’ non-parametric test under the alternative hypothesis of mean or median differences between dasometric variables and precipitation components. If differences were found, they were evaluated using the Significant Minimum Difference (DMS) method for multiple comparisons or the Mann-Whitney’s test, if non-parametric analysis was required. Significance level was set at α = 0.05 [29, 30].

## Results

The number of trees per ha and basal area before treatment were not significantly different among plots (Number of trees: F = 0.432, p = 0.734); basal area: (F = 0.208, p = 0.889). After applying the different types of cutting intensities, plots did show significant differences (Number of trees: F = 16.755, p<0.001; basal area F = 13.346, p< 0.01). As derived from Table 1, total harvested volume was 0, 67.75, 143.82, and 152 m^3^ ha^-1^, for the control group, conservative, semi-intensive, and intensive managements. Meanwhile, Shannon-Wiener’s diversity index was 1.62, 1.54, 1.44, and 0, respectively.

**Fig 3:**
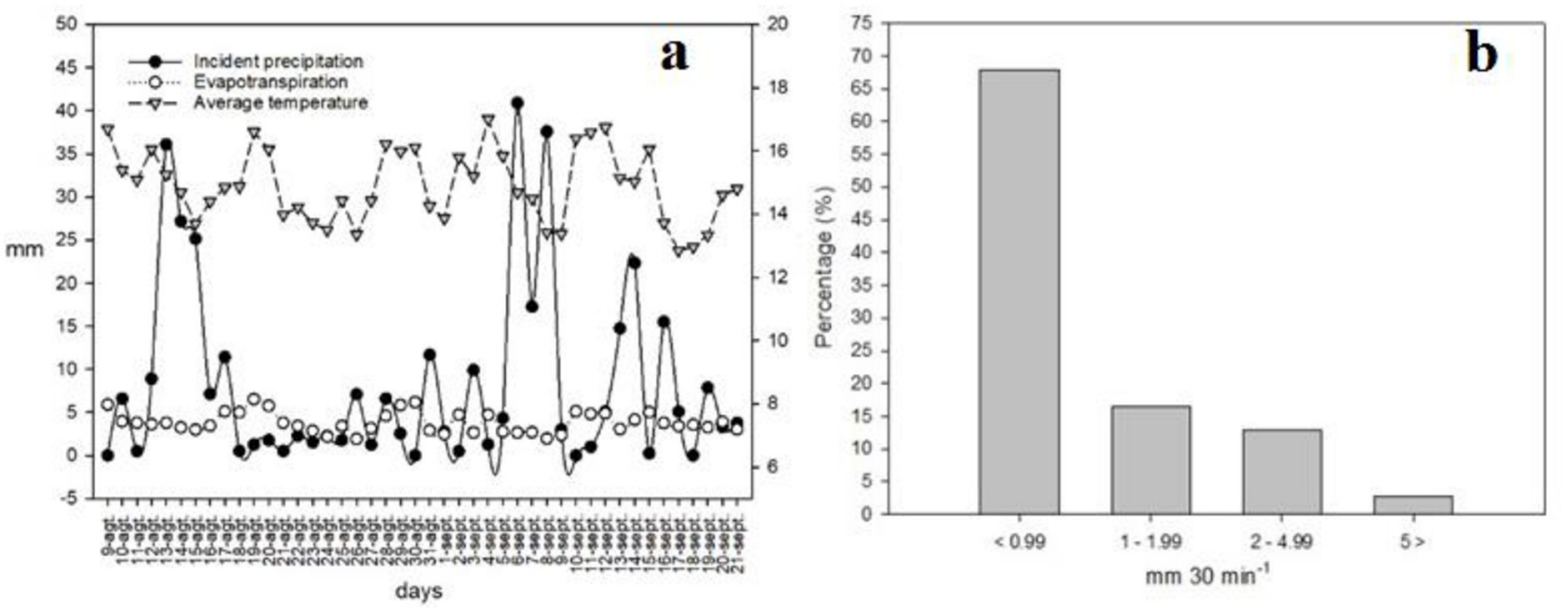
(a) Incident precipitation, evapotranspiration, and temperature measurements, (b) intensity intervals of rainfall events from August to September 2016 in a temperate forest in Durango, Mexico.

During the research period, the weather station recorded a total precipitation of 361 mm, a mean temperature of 15.8°C, and evaporation of 172.5 mm (47%), which indicated a positive hydrological balance for that period. Regarding rainfall intensity, 69% of the plots had a precipitation of less than 1 mm over a 30-minute event, 16% between 1 and 2 mm, 12.9% between 2 and 5 mm, and 2.7% greater than 5 mm for each 30-minute period (Fig 3).

### Throughfall

Throughfall (*T*) ranged between 240 to 335 mm, with 3.2 to 69 mm per rainfall event. Forty percent of the rainfall events recorded less than 20 mm, 42% between 20.1 and 40 mm, and 18% were greater than 40 mm (Table 2). We modeled the behavior of *T* in relation to *BA* using a linear equation (p = 0.0001, R^2^ = 0.85) observing that for each unit increase in *BA*, throughfall decreased by 3.03 mm (30,303 l ha^-1^) (Table 6). Throughfall events were small, only two of them reached above 60 mm.

**Table 2:** Throughfall per basal area in a temperate forest ecosystem in Durango, Mexico.

Basal area m^2^ ha^-1^	Silvicultural treatment	Throughfall or crown precipitation*			
		Event (mm)		Total (mm)	
		Min.	Max.	mm	L/ha
**0**	INT*	18.75	63.5	335.25	3 352 250
**0**	INT*	18.63	63.75	332.38	3 323 750
**5.70**	SEMI	4.85	69.00	316.18	3 161 750
**9.44**	SEMI	5.75	65.75	288.63	2 886 250
**17.36**	CON	5.00	63.13	285.00	2 850 000
**20.99**	CON	3.20	59.25	233.65	2 336 500
**25.01**	NT	4.25	61.00	255.25	2 552 500
**32.38**	NT	4.38	66.25	240.25	2 402 500

* INT: intensive management (clearing of 100% of *BA*), SEMI: semi-intensive management (cutting of 60% of *BA*), CON: conservative management (cutting of 30% of *BA*), NT: No treatment (0% cutting).

### Stemflow

**Fig 4:**
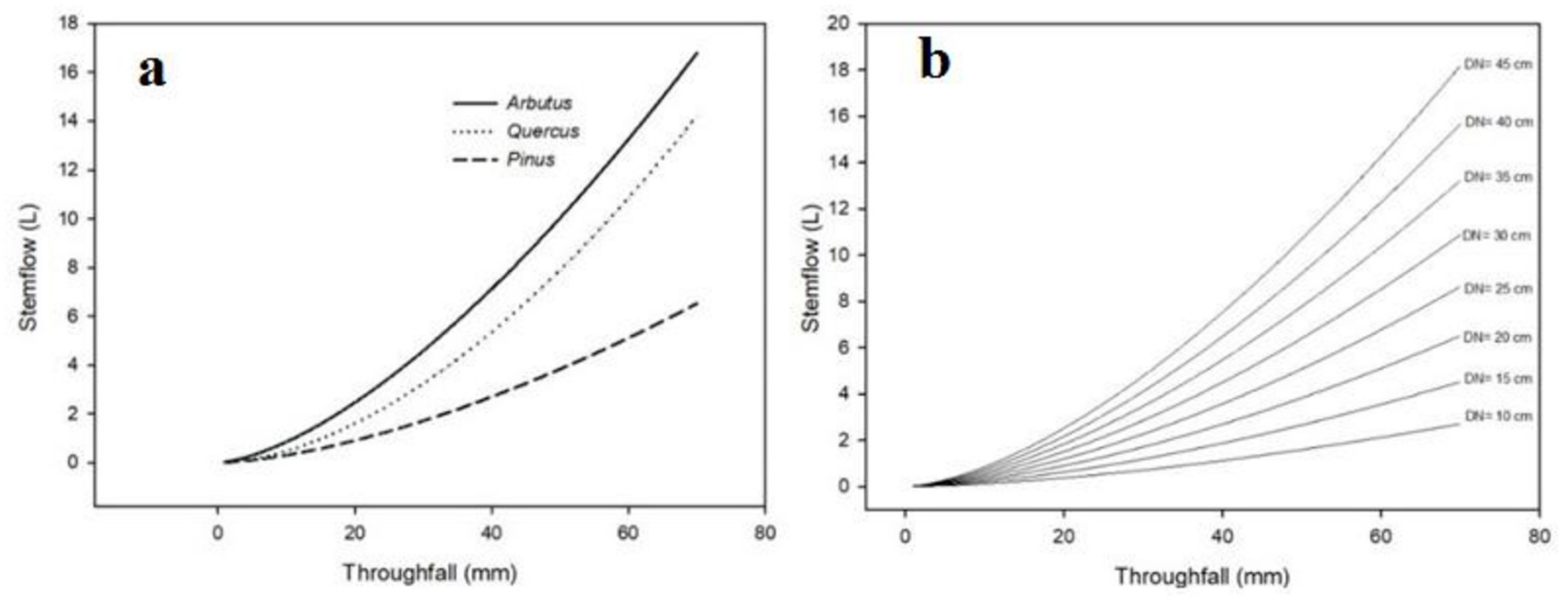
Adjusted curves of stemflow related to (a) throughfall on trees with 20 cm normal diameter per genus, and (b) throughfall with varying normal diameters for pine trees.

Stemflow (*SF*) ranged between 0.1 and 19.1 l per rainfall event in measurements where *T* ranged between 3.2 and 69 mm. The exponential models that evaluate *SF* as a function of normal diameter and throughfall for all genera were significant (p<0.001) (Table 3). The madrone tree (*Arbutus* spp.) had the highest stemflow, capturing 1.26 and 2.56 times more water than oak and pine trees. We observed that when normal diameter is constant, *SF* increases as *T* increases (Fig 4).

**Table 3:** Statistical estimates using Eq (3) to estimate stemflow per tree in a forest ecosystem in Durango, Mexico.

Genus	VAR	Parameter Estimates							
		N	Min.	Max.	Mean	SE^1^	Parameters	SE^2^	*R* ^*2*^
***Pinus***	*ND*	127	8.28	47.11	24.45	1.185	β_1_ = 0.00018	0.00009	0.86
	*T*	127	3.2	69	22.52	1.460	β_2_ = 1.2663	0.09162	
	*SF*	127	0.01	15.81	1.78	0.271	β_3_ = 1.5778	0.08170	
***Quercus***	*ND*	92	7.96	50.29	19.55	1.140	β_1_ = 0.0013	0.0007	0.81
	*T*	92	3.20	69.00	20.39	1.666	β_2_ = 0.6282	0.0850	
	*SF*	92	0.01	18.22	2.09	0.366	β_3_ = 1.7460	0.1259	
***Arbutus***	*ND*	83	7.321	27.37	14.16	0.734	β_1_ = 0.0014	0.0006	0.89
	*T*	83	4.25	69.0	23.98	1.815	β_2_ = 0.9604	0.0774	0.86
	*SF*	83	0.01	19.11	2.67	0.421	β_3_ = 1.5335	0.0978	

VAR: tree and precipitation components; *ND*: Normal diameter (cm), *T*: Throughfall in l m-^2^, S*F*: Stemflow in liters, SE^1^: standard error of mean, SE^2^: standard error of parameters.

Oaks produced the highest *SF* (54.8%), followed by pines (37.8%), and madrone trees (*Arbutus* spp.) (7.4%) due to their quantity. The highest *SF* occurred when the basal area was highest (32.38 m^2^ ha^-1^) with a total water volume of 18,782.1 l ha^-1^ (Table 4). Stemflow showed a strong positive correlation with *BA* (Pearson’s correlation coefficient = 0.99). We modeled the relationship using a linear equation with basal area (p<0.01, *R*^2^ = 0.9), which resulted that for each basal area unit increase, *SF* rose by 483 l ha^-1^ (Table 6).

**Table 4:** Stemflow by basal area and silvicultural treatments in a temperate forest ecosystem of Durango, Mexico.

*BA* m^2^ ha^-1^	Silvicultural treatment	N	Stemflow (L ha^-1^)				
			*Pinus*	*Quercus*	*Arbutus*	Total	mm
**5.70**	SEMI	31	3 385.9	1 711.8	583.5	5 681.1	0.57
**9.44**	SEMI	42	3 848.4	4 104.7	295.1	8 248.1	0.83
**17.36**	CON	33	2 919.3	4 288.6	3 930.3	11 138.2	1.11
**20.99**	CON	74	6 437.4	6 130.7	373.0	12 941.1	1.29
**25.01**	NT	70	4 672.3	10 799.6	155.8	15 627.8	1.56
**32.38**	NT	83	6 141.2	12 640.9	0.00	18 782.1	1.87
**TOTAL**			27 404.4	39 676.3	5 337.7	72 418.4	7.25

INT: Intensive management (clearing of 100% of *BA*), SEMI: Semi-intensive management (clearing of 60% of *BA*), CON: Conservative management (clearing of 30% of *BA*), NT: No treatment (0% clearing). The last column shows stemflow values after being converted to depth equivalents (mm).

### Surface runoff

During the evaluation period, *SR* ranged between 14,817.5 to 115,833 l ha^-1^, with a basal area between 0 and 32.38 m^2^ ha^-1^. We observed that *SR* increases as throughfall increases; by contrast, *SR* decreases as the basal area increases (Table 5). When modeling the relationship between *SR* and the *SR* coefficient for the period under evaluation, the best fit was obtained using an exponential equation using the basal area as an independent variable (p<0.01) (Table 6).

**Table 5:** Surface runoff per basal area in a forest of the temperate-cold ecosystem in Durango, Mexico.

Basal area m^2^ ha^-1^	Silv. Treat.	*T* mm	*CC %*	*Slope %*	*Soil text*	Runoff				
						Event (mm)		Total for the period		SR Coeff.%
						Min.	Max.	mm	L ha^-1^	
**0**	INT	335.25	0	42	-	0.478	2.216	11.583	115 833.12	3.46
**0**	INT	329.50	0	33	FA	0.077	0.489	2.349	23 490.62	0.71
**5.70**	SEMI	316.18	27.6	43	FA	0.024	1.210	4.126	41 256.87	1.35
**9.44**	SEMI	288.63	39.9	51	FA	0.039	0.822	2.660	26 596.87	0.92
**17.36**	CON	285.00	67.8	49	FA	0.026	1.068	2.795	27 953.75	0.99
**20.99**	CON	233.65	84.4	55	FA	0.043	0.358	1.352	13 518.75	0.56
**25.01**	NT	255.25	114.7	55	FA	0.038	0.304	1.449	14 494.37	0.57
**32.38**	NT	240.25	194.6	60	AL	0.030	0.428	1.482	14 817.50	0.62

CC: Crown Cover, Soil Text: Soil texture (FA: sandy loam and AL: silt clay soil), T: Throughfall, INT: Intensive management (clearing of 100% of *BA*), SEMI: Semi-intensive management, CON: Conservative management, NT: No treatment.

**Table 6:** Statistical estimated values for throughfall, stemflow, and runoff coefficient in Durango, Mexico.

Model	Parameter	SE	t	p > t	R^2^-adj	p > F
***T*** = β_0_ **+** β_1_ ***BA***	β_0_ = 326.757	11.017	29.65	0.0001	0.82	0.0001
	β_1_ = -3.030	0.5787	-5.236	0.0034		
***SF*** = β_0_ **+** β_1_ ***BA***	β_o_ = 3144.32	409.51	7.671	0.002	0.99	0.0001
	β_1_ = 483.047	19.91	24.25	0.0001		
***SR coeff*. = *exp (***β_0_ **+** β_1_ ***BA)***	β_0_ = 0.738	0.243	3.038	0.029	0.689	0.0020
	β_1_ = -0.048	0.013	-3.783	0.013		
***SR* = *exp* (**β_0_ **+** β_1_***T*)**	β_0_ = -4.313	0.796	-5.419	0.003	0.88	0.001
	β_1_ = 0.019	0.003	6.717	0.001		

T: throughfall (mm), SF = stemflow (L ha^-1^), SR coeff.: runoff coefficient (%), SR: surface runoff (mm), *BA*; basal area (m^2^ ha^-1^); β1, β2, and β3: regression parameters.

### Interception and net precipitation

Using the models that were generated (Table 6), we estimated the values of *T*, *SF*, and *SR* for the remaining plots, and with Eqs (1) and (2), we estimated interception and net precipitation for all plots. For the purpose of this study, evaporation in the intensive treatment (clearcutting) was assumed to be part of interception. Overall, prompted by interception or evaporation, the value of net precipitation (*NP)* increases as *BA* increases. Regarding the values of *NP*, *I*, and *SR*, the silvicultural treatments were statistically different (Net precipitation: F = 8.186, p = 0.004_;_ Interception: F = 8.493, p = 0.003, surface runoff: F = 3.282; p = 0.062) (Table 7). Mean surface runoff was larger in the intensive treatment and interception was higher in the control group.

**Table 7:** Measures of throughfall, stemflow, net precipitation, interception, and surface runoff for each forest management type examined in Durango, Mexico.

Variable	Mean	Min	Max	Std Dev	%*
	**Control Group (NT)**				
Basal area (m^2^ ha^-1^)	23.2	17.0	32.4	7.0	
Throughfall (mm)	260.4	240.3	275.4	16.0	72.3
Stemflow (mm)	1.4	326.8	335.3	4.3	0.4
Net precipitation (mm)	261.9	242.1	276.5	15.6	72.7
Interception (mm)	98.7	84.6	117.9	15.0	27.4
Surface runoff (mm)	1.9	1.5	2.6	0.6	0.5
	**Conservative Management (CON)**				
Basal area (m^2^ ha^-1^)	14.5	7.4	21.0	5.9	
Throughfall (mm)	278.2	233.7	304.2	30.8	77.3
Stemflow (mm)	1.0	0.7	1.3	0.3	0.3
Net precipitation (mm)	279.2	234.9	304.9	30.6	77.5
Interception (mm)	81.2	55.8	125.1	30.3	22.5
Surface runoff (mm)	3.0	1.4	4.5	1.3	0.8
	**Semi-intensive management (SEMI)**				
Basal area (m^2^ ha^-1^)	8.7	5.7	11.9	2.6	
Throughfall (mm)	299.8	288.6	316.2	12.8	83.3
Stemflow (mm)	0.7	0.6	0.9	0.2	0.2
Net precipitation (mm)	300.6	289.5	316.8	12.6	83.5
Interception (mm)	59.8	43.3	70.6	12.8	16.6
Surface runoff (mm)	3.7	2.7	4.4	0.8	1.0
	**Intensive management (INT)** **				
Net precipitation (mm)	330.5	362.8	335.2	4.33	91.5
Surface runoff (mm)	6.92	2.35	11.6	4.62	1.9

* Percentage relative to incident precipitation (361 mm);

** No values are reported for basal area, throughfall, stemflow, and interception (clearcutting treatment).

In the intensive management, net precipitation includes rain that reaches the ground directly.

In addition, the effect of cutting on net precipitation (*NP)* and interception (*I)* in the control group was similar to the conservative treatment, while the effect of the semi-intensive treatment on *NP* and *I* was similar to the intensive treatment. Furthermore, surface runoff (*SR)* did not show differences among the control group, conservative, and semi-intensive treatments, albeit they were significantly different when compared to the intensive management type. Average residual basal areas of 23.21, 14.5, 8.65, and 0 m^2^ ha^-1^ were observed in the control group, conservative, semi-intensive, and intensive managements, respectively. Net precipitation, defined here as the proxy for water yield, was 2.62, 2.79, 3.0, and 3.31 million of l ha-^1^, respectively. The losses due to interception or evaporation were higher for the control group and lower for the semi-intensive treatment (Table 7).

**Fig 5:**
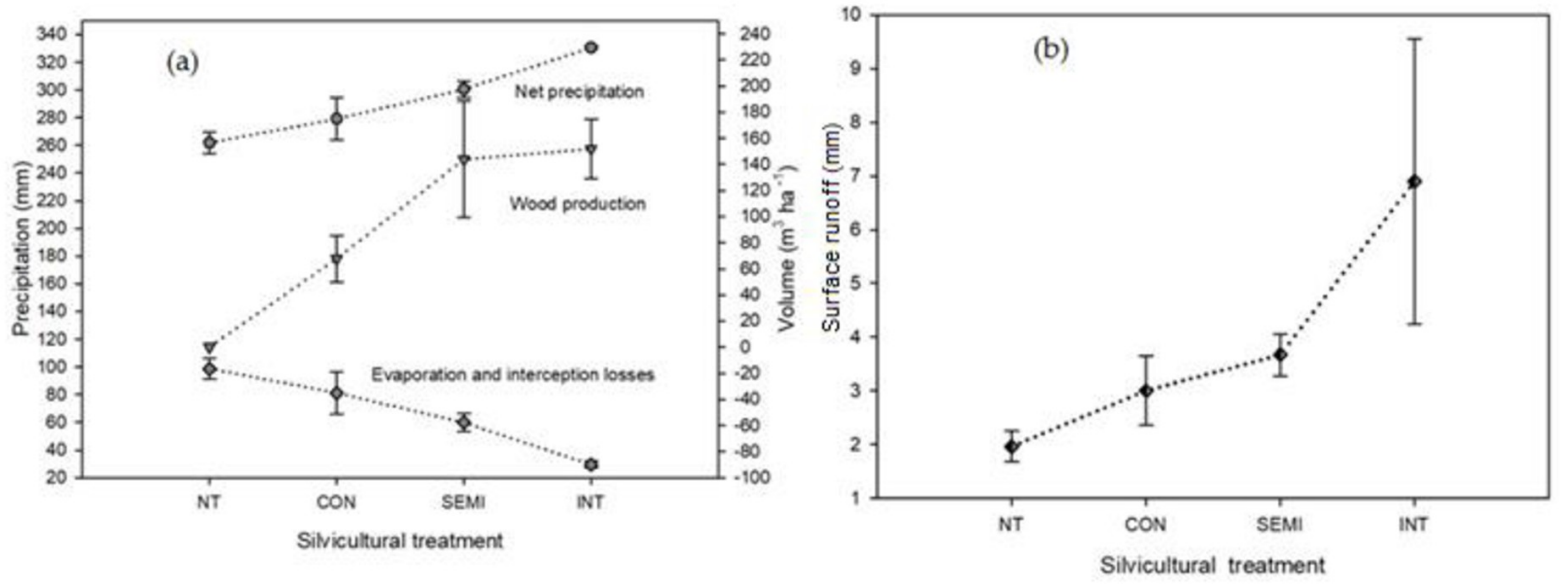
Relationship between timber harvesting and interception and evaporation losses (a) and relationship between surface runoff and silvicultural treatments (b).

The highest water yield, highest timber availability, lowest diversity, and lowest interception or evaporation losses were observed in management scenarios with the lowest tree cover (*SEMI* and *INT*). Notwithstanding, these had the highest surface runoff and therefore, greater conditions for hydrological erosion. In contrast, the conservative management scenario had the lowest surface runoff, which in turn minimizes erosion due to loss of vegetation cover, eventually allowing timber harvesting. In addition, species diversity remained constant and water yield increased during the study period (Fig 5).

## Discussion

The objective of this study was to evaluate the effects of various silvicultural treatments on water flow in the forest canopy and the soil. The treatments were based on the residual basal area that resulted from the application of clearcutting and individual selection of trees. All silvicultural treatments, except for the control group, showed an increase in water yield, particularly surface runoff. Those that received the higher percentage of *BA* cutting (conservative, semi-intensive, and intensive) had the highest water yield. When *BA* decreased from 23.21 to 14.5 m^2^ ha^-1^ it caused a 2 mm increase per *BA* unit in surface runoff. When *BA* was reduced from 14.5 to zero, the change per *BA* unit was 3.4 to 3.6 mm. According to Hornbeck et al. [22], the increase in water availability is proportional to the percentage reduction of basal area. An oak forest with a 50% reduction of *BA* showed a 13.2 mm increase in water availability (4.6%), while clearcutting increased water availability by 42.8 mm (14.7%). Bosch and Hewlett [5], found that a 10% change in *BA* would cause a reduction of 40, 25, and 10 mm in water yield in eucalyptus, deciduous, and scrubland forests respectively. Contrarywise, Mediterranean forests with 18% removal did not show increase in water yield during the first and second year after cutting [31].

Cruz-Garcia, et al., found that the minimum *SR* values were observed in forests with a basal area higher than 20 m^2^ ha^-1^ [23]. Rivera-Ruiz, et al., [32] in a river basin in central Mexico, found values of 0.03%, 0.09%, 2.14%, and 4.92% *SR* for forest plantations with pine, native grassland, grass, and soil without vegetation, respectively. Another study in central Mexico, with pine and oak forests, reported the results of surface runoff for two conditions, which both had an annual incident precipitation of 1,070 mm. In the first case, *SR* yielded 0% while the second registered 4.6% (49.5 mm) [33]. The difference between these two conditions was that the former had considerable tree cover, while the latter’s topsoil had almost disappeared and was composed of some herbaceous and bushes [34]. Elsewhere, in watersheds composed of broad-leaf and mixed forests, a change in 1% forest cover resulted in a change of 0.80% and 0.74% in annual runoff, respectively; while in watersheds dominated by large conifer forests, the change was just 0.24% [35].

The values of *SR* observed in this study were small, possibly because rainfall events were too small to produce surface runoff (85% had less than 2 mm in a 30-min event). According to Viramontes et al., [33, 34], the minimum rainfall intensities that create runoff must be greater than 20 mm hr^-1^. The areas evaluated in this study had a well-established herbaceous cover with up to 6 cm of topsoil and arrangement of residual material. In addition, in the intensive treatment, owners applied subsoiling, following contour lines, and dispersed mulched material, which favored water infiltration and slightly decreased surface runoff. Apparently, having a thick topsoil layer and vegetation cover helps reduce the effect of texture and slope on runoff volume [33].

Overall, our study was consistent with those that have reported increases in surface runoff following forest harvest treatments [23, 24]. Changes in runoff were greater following larger reductions in basal area. However, the changes we documented were less than described in Hornbeck et al. [22] and Bosch and Hewlett [5], but larger than those reported for Mediterranean forests. Some studies even suggest that to have noticeable increases in water yield, vegetation cover must be reduced by 20% [5, 36–38]. This is because the loss of tree cover produces more surface runoff and eventually increases the risk of soil erosion [37, 38].

Our study is also consistent with others reporting a decrease in interception loss associated with a reduction in basal area [37]. Removal of 30% basal area caused an average of 34.3% decrease in interception, whereas it decreased by 17.51%, 21.41%, and 30.31% in the conservative, semi-intensive, and intensive management scenarios, respectively. Breda et al., [39], reported that in an oak forest that had 35% of basal area removed in the first year, showed an interception decrease of 16% in the treatment plot, while the control plot had 23%. In the second year, interception was 17% in both plots [38]. Bäumler and Zech [40] found that the removal of 40% of the forest volume, produced a decrease in interception of up to 45%. Also, Aboal et al., [41] showed that cutting 15% of *BA* in a pine forest, reduced interception by 11.1% (36). Similarly, when comparing treatments with no cutting, 50% cutting and 100% clearing of *BA*, interceptions registered 9.0%, 6.7%, and 1.8%, respectively [42].

In the silvicultural treatments that retained vegetation cover, throughfall ranged from 72.3% to 83.28% related to incident precipitation, stemflow varied from 0.2% to 0.4%, and interception ranged from 16.61% to 27.42%. In this regard, the conservative scenario (30% cutting, *BA* of 14.5 m^2^ ha^-1^) and the control group (0% cutting, *BA* of 23.21 m^2^ ha^-1^) treatments had the least throughfall and stemflow, while interception was the largest. Similar results were seen in a study of water balance in oak-pine forests in Michoacan, Mexico that had an 895 mm incident precipitation. They found that interception was 13%, stemflow was 1.9%, and throughfall was 87% [43]. In Nuevo Leon, Mexico, also in pine-oak forests, the interception was on average 18.6%, while stemflow was 0.22%, over incident precipitation [44]. Similarly, in pine-oak forests in San Luis Potosi, Mexico, with 1,188 mm of incident precipitation, throughfall and stemflow were 15% and 20% greater, respectively, in oak forests compared to only pine forests [45]. In a different ecosystem, i.e., a thorny scrubland, with a 488 mm of incident precipitation, throughfall ranged from 76% to 85%, stemflow ranged between 1.2% and 3%, and interception varied between 13% to 22% [11, 46]. The variety of results of these studies suggests that precipitation components differ from local (i.e., slope, soil texture, species composition, etc.) and regional conditions (precipitation, evapotranspiration), but, overall, vegetation highly influences the magnitude of each component. Ellison *et al*., after reviewing several papers in global hydrology, concluded that forests should be considered as water suppliers rather than water consumers, contrary to what was perceived for many years [47].

Among the limitations of the study are that a baseline from which basal area reduction starts to increase water yield was not defined. Thus, additional long-term, year-to-year studies are needed to monitor water flow as vegetation cover recovers, and to consider various conditions such as changes in tree structures and cutting intensities. Other factors to explore include soil preservation practices and topographic characteristics, like slope, soil type, and site quality. In addition, studies that seek to optimize maximum water yield and greater timber harvest in relation to other ecosystem services, such as biodiversity conservation and climate regulation, are needed.

## Conclusions

The results of this study contribute to the knowledge of the impact of forest management treatments on water balance components in a temperate forest in northern Mexico. These components heavily depend on vegetation cover, which in turn depend on the type of silvicultural treatments. Changes in vegetation cover, in terms of basal area, modified water yield and rainfall distribution. Intensive management scenarios registered the highest surface runoff compared to the conservative and the control group treatments. The study found that a unit increase in basal area (m^2^ ha^-1^) led to a reduction of 2 to 3.6 mm in surface runoff.

Surface runoff was small during the evaluation period, compared to other studies in Mexico. We believe it was due to the existing understory vegetation, low rainfall intensity, and complementary practices supporting soil conservation, such as subsoiling. This underlines the importance of applying such practices to increase the amount of water that infiltrates into the soil. The conservative management scenario is a viable alternative for increasing water yield, maintaining species’ composition, and minimizing the risk of soil erosion. This is because of the low loss of vegetation cover and less surface runoff. There is trade-off between this management scenario and timber production due to the low cutting intensity, but it heavily favors other ecosystem services. This study can help resource managers understand the interactions between forest management and water yield, improve decision making, and prepare sustainable forest management plans.

## Supporting information

S1 Data (XLSX)
